# First Sternal Cleft Repair Using a Porous Alumina Ceramic Prosthesis in a 9-Year-Old Child

**DOI:** 10.1055/s-0039-1688775

**Published:** 2019-08-26

**Authors:** Virginie Fouilloux, François Bertin, Emilie Peltier, Jean-Luc Jouve

**Affiliations:** 1 Department of Cardiac Surgery, Timone Children's Hospital, Marseille, France; 2 Faculty of Medicine, Aix-Marseille University, Marseille, France; 3 Department of Thoracic and Vascular Surgery, Dupuytren University Hospital, Limoges, France; 4 Department of Orthopaedics, Timone Children's Hospital, Marseille, France

**Keywords:** sternal cleft, prosthesis, bioceramics, tissue engineering

## Abstract

Sternal cleft is a rare congenital abnormality, often associated with other congenital defects. We present the case of a 9-year-old child with complete sternal cleft, treated with an innovative sternal prosthesis. Surgery was performed to protect the heart and also, as pulsations was visible, leading to serious esthetical concerns, to enhance school integration, which was difficult. The porous alumina device used was initially designed for sternal reconstruction after refractory deep sternal wound infection or carcinoma. Surgery and early follow-up were simple. There was no complication and the follow-up of more than 1 year reveals a good healing without breath discomfort and a correct development of the chest wall. In this rare indication, the alumina ceramic sternal prosthesis offers a reliable alternative to classical methods, such as muscle flap, autogenous tissue transfer, costal homograft, and other prosthetic materials like mesh or synthetic patch.

## Introduction

Sternal cleft is a congenital failure of sternal fusion during the embryological development. It is often associated with other malformations, such as cardiac anomaly. 1 Several classifications have been proposed based either on the anatomical level 2 or on the extension of the cleft process. 3 4 To our knowledge, Down's syndrome is usually not associated to this type of sternal malformation. Indication of surgical closure are mainly for the protection of mediastinum and underlying organs, improvement of breathing, and to avoid recurrent respiratory infections. 5 Because of higher compliance of the thoracic cage associated to a minimal compression of underlying structures in young children, early surgery is usually preferred. 6 However, when sternal cleft closure has been delayed, the best approach for surgical treatment remains unclear. A lot of clinical cases using various surgical technics are reported, but it is difficult to draw conclusion about the gold standard technique. 1 5

Bioceramics were originally used as vertebra cages and for complex orthopaedic procedures. Recently, a ceramic sternal prosthesis was designed to replace sternum when removed for tumor or infectious reasons. 7 We describe this new surgical option to fix this sternal defect.

## Case Report

A 9-year-old girl (height = 120 cm, weight = 16 kg) with Down's syndrome was referred to our institution with a butterfly-shaped congenital sternal cleft. The patient had been operated for a congenital heart defect (cor triatriatum) in the 1st week of life which left this unhealed congenital sternal cleft. As often in neonatal cardiac surgery, chest closure was delayed a few days and then, sternal rims were just sealed and the cleft was left open because of the cardiac surgery.

Cardiac echocardiogram was normal at readmission. A midline defect with visible chest pulsations caused serious esthetical concerns and hard school integration ( Video 1 ). Because of intellectual impairment related to Down's syndrome, respiratory function testing was not available.

**Video 1**

Preoperative visit.

**Fig. 1:**
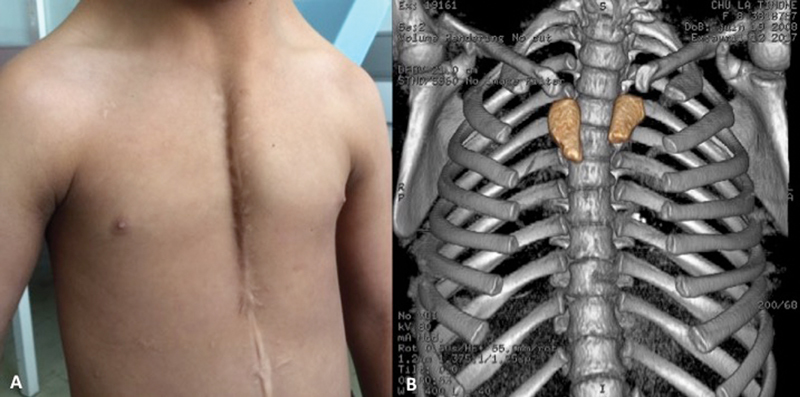
( **A** ) Preoperative view; ( **B** ) preoperative CT-scan 3D reconstruction. 3D, three-dimensional; CT, computed tomography.

As shown in Fig. 1A , the previous cardiac surgery scar went on a skin raphe, as sometimes described in this rare congenital defect (failure of midline development). Clinical findings were confirmed by chest tomodensitometry ( Fig. 1B ). To preserve chest wall stability, optimize growth evolution, and avoid some pitfall of other techniques, we choose a ceramic prosthesis (Sternum Ceramil, I.Ceram, Limoges, France) for surgical repair. As at this time, the European Certification was not yet obtained, the surgery was performed thanks to a waiver from the French Agency for Health Security (ANSM) and the patient's parents who gave their consent for this surgery. Since this surgery this device has been CE marked.

At surgery, prior cutaneous scar was excised as much as possible. Pectoralis major muscle was elevated on either side from their sternal origin.

**Fig. 2:**
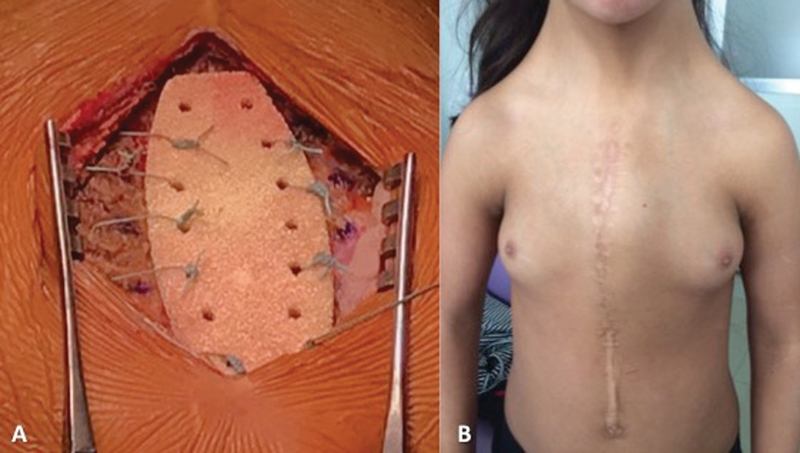
( **A** ) Peroperative view of I.Ceram porous alumina sternal prosthesis insertion with sutures; ( **B** ) postoperative view (month 12).

Costal cartilages were gently and softly excavated to leave space for prosthetic sternum implantation. This stage was simplified using the trial implant which allowed removing only the necessary amount of cartilages. The trial implant has the same shape and size as the ceramic but is in stainless steel. It is used to choose the size and to prepare the area of implantation. The preperforated prosthetic ceramic sternum (size 1) was anchored to cartilages with eight nonabsorbable 3/0 polyester sutures ( Fig. 2A ). Closed suction drains were placed above and below the sternum. The pectoralis major muscles were approximated medially and the skin was closed with subcutaneous and cutaneous continuous sutures.

Chest drains were removed on day 3 and the patient was discharged on day 7. Early and long-term postoperative pain was limited. At the last follow-up visit (month 12), the patient was doing well and the scar was totally healed, clean, and dry ( Fig. 2B , Video 2 ), as it was since the first outpatient visit at day 10.

**Video 2**

Post operative visit (month 6).

## Discussion

Cleft sternum is a rare congenital abnormality, less than 1 among 100,000 births, in which there is no sternal bone fusion.

Surgery is not mandatory 8 but it reduces the risk of respiratory infections, paradoxical respiratory movement, damages of mediastinum, and organs.

Three main surgical strategies have been described for congenital cleft closure: (1) direct closure, (2) closure using autologous tissue, or (3) artificial material. 1 5 8 9 However, no gold standard has emerges to date as the number of cases is very small.

When surgery is performed early, device-free surgical approach is recommended with approximation of sternal wedges. After 3 months, elasticity of thoracic cage decreases and direct closure might be hazardous. It is difficult to say whether an early surgery is better than a delayed one as no trial compared these two options. However, in our case, as an early surgery was not possible due to the cardiac surgery, the second option was mandatory.

In older patients, the use of various autografts (ribs, costal cartilage, etc.) or prosthetic materials (titanium plates, silicone, etc.) has been described. 1 4 5 9 These techniques may lead to residual pain and exposes patients to infectious complications. Seromas have also been frequently seen. Harvesting bone graft can be challenging and using mesh can result in a chest instability and paradoxical motion. 5

This prosthesis is a porous ceramic made of pure alumina (Al _2_ O _3_ ). It is fully biocompatible and nonabsorbable. This alumina sternum has been implanted in adults 11 times before this surgery. These surgeries were performed in other hospitals in France for adult patients with a mean age of 58 years (range, 38–79 years). 7 Indications were sternal cancer involvement ( *n* = 8) or refractory deep sternal wound infection ( *n* = 3). At the time of this surgery, the mean follow-up of these patients was 18 months with the older one dating from 3 years. No complications were observed.

Bioceramic provides a new approach for tissue engineering. Porous characteristics (with pores mainly ranging from 100 to 900 µm) allow attachment of osteoblasts and chondroblasts to the ceramic. Its biocompatibility has been well demonstrated with more than 5,000 implantations of cervical cages and tibial wedges. 10 11 The risk of infection is lower than with metal. 12 13 Long-term follow-up showed no case of local/systemic effect and alumina ceramics are classified as inert. 14

The resistance of the ceramic while compressed is superior to 20 MPa (thrice the one of cancellous bone). Its ovoid shape is particularly adapted and five different sizes are routinely available associated to three half shape to replace only the manubium part.

For those attractive reasons and the adult experience, 7 we decided to try this new material in this rare condition. The rigidity of the ceramic with the smooth anchorage using suture threads avoids chest instability while giving elasticity. We hypothesized that this porous alumina ceramic could provide a good long-term option, with a preserved growth potential. Indeed, preserving the rib cartilages will allow a growth of the thoracic cage.

Finally, we deplore the final aspect of the scar, which resulted from the first neonatal surgical procedure, and the delayed chest closure.

## Conclusion

Thanks to an easy surgical technique and a long follow-up (> 12 months) the use of the I.Ceram porous alumina sternal prosthesis is a good option in this rare congenital anomaly.

## Notes

New Insights and the Importance for the Pediatric Surgeon We present an innovative technique for sternal cleft closure, using a porous alumina prosthesis. This is the first use in this indication. Outcomes are excellent.
